# Stimulator of Interferon Genes Promotes Host Resistance Against *Pseudomonas aeruginosa* Keratitis

**DOI:** 10.3389/fimmu.2018.01225

**Published:** 2018-06-05

**Authors:** Kang Chen, Qiang Fu, Siping Liang, Yiting Liu, Wenting Qu, Yongjian Wu, Xinger Wu, Lei Wei, Yi Wang, Yujuan Xiong, Weijia Wang, Minhao Wu

**Affiliations:** ^1^Department of Laboratory Medicine, Zhongshan Hospital of Sun Yat-sen University, Zhongshan, China; ^2^Program of Pathobiology and Immunology, Fifth Affiliated Hospital, Zhongshan School of Medicine, Sun Yat-sen University, Guangzhou, China; ^3^Guangdong Engineering & Technology Research Center for Disease-Model Animals, Sun Yat-sen University, Guangzhou, China; ^4^Department of Neurology, The Third Affiliated Hospital of Sun Yat-sen University, Guangzhou, China; ^5^Department of Laboratory Medicine, The Second Affiliated Hospital of Guangzhou University of Chinese Medicine, Guangzhou, China

**Keywords:** STING, *Pseudomonas aeruginosa*, corneal infection, inflammation, bacterial killing

## Abstract

*Pseudomonas aeruginosa* (PA) is the leading cause of bacterial keratitis, especially in those who wear contact lens and who are immunocompromised. Once the invading pathogens are recognized by pattern recognition receptors expressed on the innate immune cells, the innate immune response is stimulated to exert host defense function, which is the first line to fight against PA infection. As a converging point of cytosolic DNA sense signaling, stimulator of interferon genes (STING) was reported to participate in host–pathogen interaction. However, the role of STING in regulating PA-induced corneal inflammation and bacterial clearance remains unknown. Our data demonstrated that STING was activated in murine model of PA keratitis and in *in vitro*-cultured macrophages, indicated by Western blot, immunostaining, and flow cytometry. To explore the role of STING in PA keratitis, we used siRNA to silence STING and 2′,3′-cGAMP to activate STING *in vivo* and *in vitro*, and the *in vivo* data found out that STING promoted host resistance against PA infection. To investigate the reason why STING played a protective role in PA keratitis, the inflammatory cytokine secretion and bacterial load were measured by using real-time PCR and bacterial plate count, respectively. Our data demonstrated that STING suppressed the production of inflammatory cytokines and enhanced bacterial elimination in murine model of PA keratitis and in PA-infected macrophages. To further investigate the mechanism beneath, the phosphorylation of mitogen-activated protein kinase, the nuclear translocation of nuclear factor-κB (NF-κB) and the bactericidal mechanism were measured by western-blot, immunofluorescence, and real-time PCR, respectively. Our data indicated that STING suppressed inflammatory cytokine expressing *via* restraining NF-κB activity and enhanced inducible NO synthase expression, an oxygen-dependent bactericidal mechanism. In conclusion, this study demonstrated that STING promoted host resistance against PA keratitis and played a protective role in PA-infected corneal disease, *via* inhibiting corneal inflammation and enhancing bacterial killing.

## Introduction

*Pseudomonas aeruginosa* (PA) is the leading cause of microbial keratitis in those who are immunocompromised and contact lens users (1). Without appropriate treatment, PA keratitis can lead to a rapidly progressive corneal disease with adverse pathological tissue damage such as inflammatory epithelial edema, stromal infiltration, corneal opacification, corneal perforation, and even permanent vision loss (2). Murine models of resistant BALB/c mice (cornea heals) and susceptible C57BL/6 mice (cornea perforates) were used as animal models of bacterial keratitis, to facilitate research on corneal immune defenses against PA (2, 3).

The innate immune system is critical for efficient host defense against pathogen invasion. Invading pathogens are recognized by pattern recognition receptors (PRRs) expressed on the innate immune cells such as macrophages and neutrophils, which are recruited to the infectious local cornea. These innate immune cells initiate the production of inflammatory cytokines such as interleukin 1 beta (IL-1β), interleukin 6 (IL-6), macrophage inflammatory protein 2 (MIP-2), and tumor necrosis factor α (TNF-α) (2, 4), and meanwhile provoke bactericidal mechanisms such as reactive oxygen species (ROS) (5) and reactive nitrogen species (6). These inflammatory mediators also promote bacteria clearance, nonetheless, if uncontrolled, result in tissue damage and corneal perforation. At this moment, conventional therapies, such as antibiotic treatment, may often fail to reverse the tissue damage caused by amplified inflammation, even if bacteria were erased from the cornea. Thus, it is critical to develop new strategies to balance bacterial killing and inflammatory overreaction (2, 4).

Stimulator of interferon genes (STING), an endoplasmic reticulum (ER)-resident molecule, is a recently found PRRs and a converging point of cytosolic DNA receptors. Once cytosolic DNA or cyclic dinucleotides are recognized by DNA receptors such as cyclic GMP-AMP synthase (cGAS), STING is triggered, phosphorylated, translocated from ER to perinuclear area, and formed perinuclear puncta, leading to type I IFN transcription (7–9). It is reported that STING is involved in various pathogen infections and exerts different functions based on pathogens and different infectious models. STING was activated when infected with herpes simplex virus (HSV) (10, 11), cytomegalovirus (CMV) (12), human immunodeficiency virus (HIV) (13), and *Mycobacterium tuberculosis* (14, 15) and promoted pathogen elimination; however, STING was triggered by *Brucella* species (16) and *Staphylococcus aureus* (17) infection, but facilitated bacterial escape. In regard to infection with *Listeria monocytogenes*, STING could restrict bacterial elimination (18), as well as mediate host defense (19), according to different infectious models. However, the role of STING in PA infection remains unknown.

Previous studies elucidated that STING-induced type I IFN is critical in host defense against virus (10–13) and intracellular bacterial infection (15); however, whether the mechanism of STING in acute PA-infected corneal inflammatory disease depends on type I IFN remains doubtful. During PA infection, more than one class of PRRs including toll-like receptors (TLRs) was activated, which were the main PRRs to be involved in PA keratitis (20–24). The interference of innate signaling ensured an effective host response (25, 26). Sharma et al. demonstrated that STING counteracted with TLR signaling and potently suppressed inflammation in a model of systemic lupus erythematosus and peritonitis (26). However, whether STING signaling regulates inflammation by counteracting with TLR signaling, including TLR downstream signal molecule mitogen-activated protein kinase (MAPK) and nuclear factor-κB (NF-κB) in PA keratitis, remains unclear.

In the present study, we demonstrated that STING was upregulated in PA-infected mouse corneas and macrophages. *In vivo* and *in vitro* silencing and activating studies indicated that STING reduced the severity of PA keratitis, by suppressed inflammation and enhanced bacterial elimination. Furthermore, we demonstrated that STING suppressed inflammatory cytokine expression *via* restraining NF-κB activity and promoted bacterial killing by enhancing inducible NO synthase (iNOS) expression. Together, these data demonstrated the beneficial role of STING in PA keratitis.

## Materials and Methods

### Ethics Statement

This study was carried out in accordance with the guidelines of Animal Care and Use of Sun Yat-sen University. The protocol was approved by Sun Yat-sen University.

### Reagents

The inhibitors of p38 (SB 203580), c-Jun N-terminal kinase (JNK, SP600125), extracellular regulated protein kinase (ERK, U0126), and NF-κB (SN50) were purchased from MedchemExpress (Monmouth, NJ, USA). Neutralizing anti-IFN-β antibody was purchased from Calbiochem (Germany).

### Animal Model and Clinical Examination

C57BL/6 mice and BALB/c mice (female, 8-week old) were purchased from the Animal Supply Center of Sun Yat-sen University. Mice were anesthetized and placed beneath a 40× magnification stereoscopic microscope. The left cornea was wounded by a sterile 25 gauge needle and then was added 5 µl bacteria suspension [containing 1 × 10^6^ colony-forming unit (CFU) of American Type Culture Collection (ATCC) 19660 PA stain]. At 1, 3, and 5 days postinfection (p.i.), mice cornea was examined, to monitor the disease process. An established scale was used to grade corneal damage (27, 28): 0, the pupil was partially or fully covered by clear or slight opacity; +1, the anterior segment was partially or fully covered by slight opacity; +2, the pupil was partially or fully covered by dense opacity; +3, the entire anterior segment was covered by dense opacity; and +4, corneal perforation.

### Cell Culture

Murine macrophage-like RAW264.7 cells (ATCC, TIB-71) were cultured in Dulbecco’s modified Eagle’s medium (DMEM), containing 10% (vol/vol) fetal bovine serum (FBS), 1% penicillin–streptomycin, and 1% l-glutamine (all purchased from Invitrogen, Carlsbad, CA, USA) at 37°C. Bone marrow-derived macrophages (BMDMs) were prepared by culturing bone marrow from the femurs and tibiae of BALB/c mice (6- to 8-week-old) in DMEM containing 10% FBS, 1% penicillin–streptomycin, 1% l-glutamine, and 10% L929 conditioned medium. Non-adherent cells were removed after 24 h and cultured for 7 days.

### Silencing STING

siRNA for mouse STING (siSTING) and the appropriate negative control (siNC) were purchased from RiboBio Co., Ltd. (Guangzhou, China). siSTING or siNC (5 μl/mouse at a final concentration of 10 µM) was subconjunctivally injected into the left eye of BALB/c mice (*n* = 5/group/time) 1 day before infection and then added topically onto the infected corneas (5 μl/mouse per time at the concentration of 10 µM at 1 and 3 days p.i.). For *in vitro* study, cells were transfected transiently with siSTING/siNC using Lipofectamine 2000 (Invitrogen) following the manufacturer’s instruction. siRNAs targeting mouse STING (5′-GAGCTTGACTCCAGCGGAA-3′) were synthesized by RiboBio.

### Activating STING

2′,3′-cGAMP (cGAMP) was purchased from Invivogen (San Diego, CA, USA). cGAMP (5 μl/mouse at a final concentration of 5 µg) was subconjunctivally injected into the left eye of C57BL/6 mice (*n* = 5/group/time) 1 day before infection and then applied topically onto the infected corneas (5 μl/mouse per time at the concentration of 5 µg at 1 and 3 days p.i.). For *in vitro* study, cells were transiently transfected with cGAMP using Lipofectamine 2000 (Invitrogen) following the manufacturer’s instruction.

### Real-Time PCR

TRIzol (Invitrogen) was used to isolate total RNA from individual corneas or cell pellets. cDNA was reversely transcribed from total RNA and then amplified using SYBR Green Master Mix (Bio-Rad, Hercules, CA, USA) according to the manufacturer’s instruction. Real-time PCR primer sequences of IL-1β, IL-6, MIP-2, TNF-α, STING, IFN-β, interferon-stimulated gene 15 (ISG15), iNOS (an important isoform of NO synthase stimulated by inflammatory mediators), nicotinamide adenine dinucleotide phosphate oxidase 2 (NOX2, an important enzyme for ROS production), and β-actin are listed in Table 1. Real-time PCR was performed by using the CFX96 Real-Time PCR System (Bio-Rad). Relative mRNA levels were calculated after normalization to β-actin.

**Table 1:** Nucleotide sequence of the specific primers used in PCR amplification.

Gene	Primer sequence (5’-3’)	
β-Actin	GAT TAC TGC TCT GGC TCC TAG C	F
	GAC TCA TCG TAC TCC TGC TTG C	R

Interleukin-6	CAC AAG TCC GGA GAG GAG AC	F
	CAG AAT TGC CAT TGC ACA AC	R

Interleukin 1 beta	CGC AGC AGC ACA TCA ACA AGA GC	F
	TGT CCT CAT CCT GGA AGG TCC ACG	R

Tumor necrosis factor α	CAC AGA AAG CAT GAT CCG CGAC	F
	TGC CAC AAG CAG GAA TGA GAA GAG	R

Macrophage inflammatory protein 2	TGT CAA TGC CTG AAG ACC CTG CC	F
	AAC TTT TTG ACC GCC CTT GAG AGT GG	R

Stimulator of interferon genes	ATT CCA ACA GCG TCT ACG AG	F
	GCA GAA GAG TTT AGC CTG CT	R

IFN-β	TTC CTG CTG TGC TTC TC	F
	CAT CTT CTC CGT CAT CT	R

Interferon-stimulated gene 15	ACT AAC TCC ATG ACG GTG TCA G	F
	GTT CCT CAC CAG GAT GCT CAG	R

Inducible NO synthase	CTA AGA GTC ACC AAA ATG GCT CCC	F
	AGA CCA GAG GCA GCA CAT CAA AGC	R

Nicotinamide adenine dinucleotide phosphate oxidase 2	TCC GTA TTG TGG GAG ACT GG	F
	AAA GGG CGT GAC TCC AAT C	R

### Western Blot

To detect the corneal expression of cGAS, phosphorylated STING (P-STING), and STING, corneas (*n* = 5/group/time) were pooled from normal and infected eyes at 1, 3, and 5 days p.i. Corneas were lysed and homogenized in lysis buffer containing1 mM dithiothreitol, 1 mM phenylmethylsulfonyl fluoride, and 1% (vol/vol) protease inhibitor cocktail (Sigma, St. Louis, MO, USA). To detect the expression of STING, phosphorylated p38 (P-p38), p38, phosphorylated c-Jun N-terminal kinase (P-JNK), JNK, phosphorylated extracellular regulated protein kinases (P-ERK), and ERK in cells, the cells were lysed in the same lysis buffer. The lysis was centrifuged to pool the supernatant, and the protein concentration of the supernatant was measured by using Quick Start Bradford protein assay (Bio-Rad). Samples were loaded, separated on 10% SDS-PAGE, and then transferred to polyvinylidene fluoride membrane (Pall Life Sciences, Ann Arbor, MI, USA). Blots were blocked, incubated with cGAS primary Abs (1:1,000, Cell signaling, Carlsbad, CA, USA), P-STING primary Abs (1:1,000, Cell signaling), STING primary Abs (1:1,000, Cell signaling), P-p38 primary Abs (1:1,000, Cell signaling), p38 primary Abs (1:1,000, Cell signaling), P-JNK primary Abs (1:1,000, Cell signaling), JNK primary Abs (1:1,000, Cell signaling), P-ERK primary Abs (1:1,000, Cell signaling), and ERK primary Abs (1:1,000, Cell signaling) at 4°C overnight, and then incubated with horseradish peroxidase-conjugated secondary Abs at room temperature for 1 h. Finally, blots were visualized with Plus-ECL (PerkinElmer, Shelton, CA, USA) following the manufacturer’s instruction. The relative density values of each band were calculated by normalizing to β-actin, after detected by Adobe Photoshop 7.0 software (Adobe Systems, Inc., San Jose, CA, USA).

### Immunostaining and Hematoxylin–Eosin (HE) Staining

For immunohistochemical staining, normal and 3-day infected eyes from C57BL/6 and BALB/c mice were enucleated (*n* = 3/group/time), immersed using ice-cold PBS, embedded in Tissue-Tek OCT compound (Miles, Elkhart, IN, USA), and frozen in liquid nitrogen. Immunohistochemical staining was performed with the UltraSensitive SP Immunodetection Kit (Maixin, Inc., Fuzhou, China) following the manufacturer’s protocol. Primary antibodies (rabbit anti-mouse STING) were purchased from PeproTech (Rocky Hill, NJ, USA). Controls were similarly treated, but the primary antibody was replaced with isotype-matched goat IgG. For immunofluorescent staining, cells were seeded on sterile glass cover slips, cultured overnight, and then fixed with 4% paraform (Sigma) in 4°C. Slips were sequentially incubated with rabbit anti-mouse STING Ab (1:200, PeproTech), rabbit anti-mouse NF-κB (1:200, Cell signaling), and Alexa Fluor 488-conjugated goat anti-rabbit IgG Ab (1:1,000, Millipore, Billerica, MA, USA), followed by incubation with 4,6-diamino-2-phenyl indole (1:10,000, Sigma) for nuclear staining. Controls were similarly treated, but the primary Ab was replaced with isotype-matched IgG. For histopathology, sections were HE stained as described by others (29). All sections were visualized with a Carl Zeiss microscope (Carl Zeiss, Inc., Oberkochen, Germany).

### Flow Cytometry

For corneal single cell detection, five corneas were pooled and digested in collagenase type I (Sigma). Cell suspensions were filtered, washed by ice-old PBS, and resuspended in PBS containing 2% BSA. To clarify the cell source of STING expression, cells suspensions were incubated sequentially with the following Abs: rabbit anti-mouse STING Ab (PeproTech), Alexa Fluor 488 conjugated goat anti-rabbit IgG Ab (Millipore), APC-conjugated anti-F4/80 Ab (BD Biosciences, San Jose, CA, USA), and PerCP-Cy5.5-conjugated anti-Gr-1 Ab (BD Biosciences). To determine the percentage of immune cells infiltrating in uninfected and infected corneas, cell suspensions were incubated sequentially with PerCP-conjugated anti-CD45 Ab (BD Biosciences). Flow cytometry was performed using LSRFortessa Cell Analyzer (BD Biosciences).

### Bacterial Plate Counts

Corneas from siSTING versus siNC-treated BALB/c mice (at 5 days p.i.) or from cGAMP versus control-treated C57BL/6 mice (at 5 days p.i.) were pooled (*n* = 5/group/time). The number of viable bacteria was calculated as described before (30). Briefly, individual corneas were homogenized, diluted in a series, and seeded on Pseudomonas isolation agar (BD Difco Laboratories) in triplicate. Results are reported as log10 CFU per cornea ± SEM.

### Intracellular Bacterial Killing Assay

Cells were seeded on a six-well plate and then infected with PA. After 1 h infection, cells in one well were treated with gentamicin (at the concentration of 300 µg/ml for 30 min, Sigma) to erase the extracellular bacteria, washed with ice-old PBS, and then lysed with 0.1% Triton-X. Cells in the other duplicate well were incubated for another 1 h and then lysed according to the same procedure. A series of 10-fold dilutions were plated on Pseudomonas isolation agar (BD Difco Laboratories) in triplicate. Intracellular bacterial load was reported as CFU per 10^6^ cells ± SEM.

### Statistical Analysis

The differences in clinical score between STING siRNA and siNC-treated BALB/c mice and cGAMP versus control-treated C57BL/6 mice were tested by the Mann–Whitney *U* test. Student’s *t*-test or ANOVA was used to determine the statistical significance of other assays. Analysis was performed using Prism 6.0 software.

## Results

### STING Expression in Mouse Cornea

**Figure 1:**
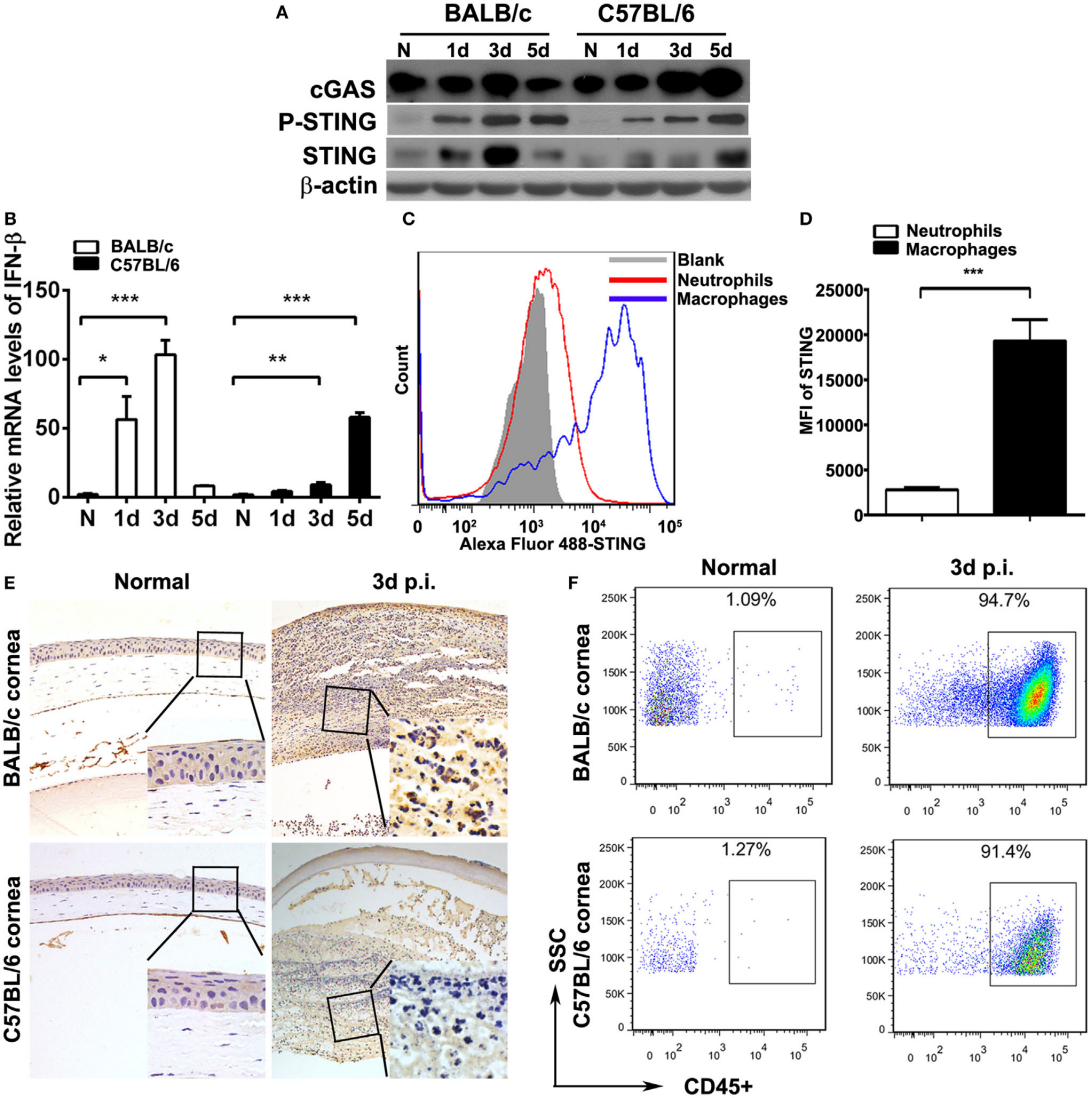
Stimulator of interferon genes (STING) expression in mouse corneas. **(A)** The expression of cyclic GMP-AMP synthase (cGAS), phosphorylated STING (P-STING), and STING was measured by Western blot in normal and infected BALB/c mouse corneas and C57BL/6 mouse corneas at 1, 3, and 5 days postinfection (p.i.). **(B)** The mRNA levels of IFN-β were measured by real-time PCR in normal and infected BALB/c mouse corneas and C57BL/6 mouse corneas at 1, 3, and 5 days p.i. **(C,D)** The expression of STING in corneal infiltrating neutrophils (Gr-1 positive) and macrophages (F4/80 positive) was measured by flow cytometry. **(E)** Immunohistochemical staining was used to detect STING expression in normal and infected BALB/c corneas and C57BL/6 corneas at 3 days p.i. (magnification = ×200). **(F)** The percentage of infiltrating immune cells in normal and infected BALB/c corneas and C57BL/6 corneas at 3 days p.i. was measured by flow cytometry. All data represent one of three independent experiments each using five pooled corneas per time. MFI, mean fluorescence. *P < 0.05; **P < 0.01; and ***P < 0.001.

To investigate STING activation during the process of PA keratitis, protein levels of cGAS, P-STING, and STING and the mRNA levels of IFN-β in mouse cornea before and after PA infection were measured by Western blot, immunostaining, and real-time PCR. Data indicated that the protein levels of cGAS, P-STING, and STING (Figure 1A) and the mRNA levels of IFN-β (Figure 1B) were first increased at 1 and 3 days p.i., and then reduced at 5 days p.i. in BALB/c mouse cornea, whereas cGAS, P-STING, STING (Figure 1A), and IFN-β (Figure 1B) expression was gradually increased at 1, 3, and 5 days p.i. in C57BL/6 mouse cornea. Immunostaining data showed that STING was not detected in normal uninfected mouse cornea (either BALB/c or C57BL/6; Figure 1E) and was mainly expressed in infiltrated inflammatory cells (Figure 1E). Meanwhile, immunostaining data also showed that STING expression in BALB/c cornea at 3 days p.i. was higher than in C57BL/6 (Figure 1E), which was consistent with the former Western blot data. To determine the percentage of immune cells in normal uninfected and infected mouse cornea, flow cytometry was applied and the data showed that approximately 1% immune cells (CD45^+^ cells) were located in normal uninfected cornea (both in BALB/c and C57BL/6 corneas, Figure 1F); however, after PA infection, immune cells were recruited and above 90% immune cells (CD45^+^ cells) were infiltrated in infected cornea (both in BALB/c and C57BL/6 corneas, Figure 1F). To clarify the cell source of STING, flow cytometry was used to determine STING expression in two major infiltrated inflammatory cells in the infected corneas, macrophages (F4/80^+^ cells), and neutrophils (Gr-1^+^ cells). The data showed that STING was mainly expressed in macrophages, rather than in neutrophils, as indicated by MFI (Figures 1C,D). These data together demonstrated that STING was activated in PA-infected mouse cornea.

### Silencing STING Accelerated the Disease Process of PA Keratitis

**Figure 2:**
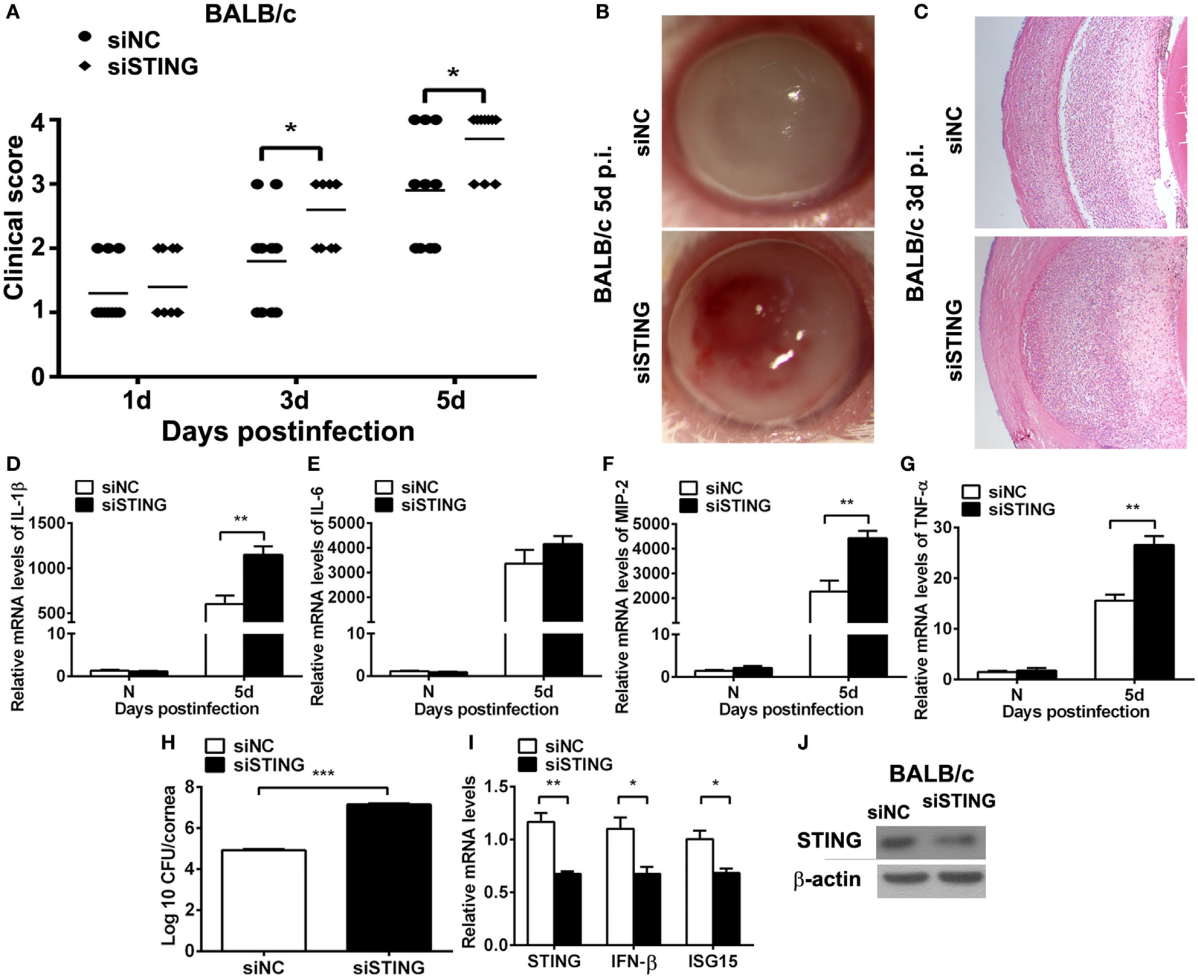
Silencing stimulator of interferon genes (STING) accelerated the disease process of *Pseudomonas aeruginosa* (PA) keratitis. BALB/c mice were subconjunctivally injected with siRNA for mouse STING (siSTING) versus siNC and then infected with PA routinely. **(A)** Clinical scores indicated the severity of the disease in siSTING- versus siNC-treated groups. **(B)** Representative slit photographs of mouse corneas at 5 days postinfection (p.i.) displayed more opacity in siSTING- versus siNC-treated mouse cornea (magnification ×10). **(C)** Hematoxylin–eosin staining was used to detect histopathology of infected cornea at 3 days p.i. in siSTING- versus siNC-treated groups (magnification ×100). **(D–G)** mRNA levels of interleukin 1 beta (IL-1β) **(D)**, interleukin 6 (IL-6) **(E)**, macrophage inflammatory protein 2 (MIP-2) **(F)**, and tumor necrosis factor α (TNF-α) **(G)** were measured by using real-time PCR in normal and infected corneas at 5 days p.i. between siSTING- and siNC-treated groups. **(H)** Bacterial load of infected cornea at 5 days p.i. was measured by plate count in siSTING- versus siNC-treated groups. **(I)** mRNA levels of STING, IFN-β, and interferon-stimulated gene 15 (ISG15) and protein levels of STING **(J)** were measured by using real-time PCR and Western blot after treatment with siSTING versus siNC. All data represent one of three independent experiments each using five pooled corneas per time. **P < 0.01 and ***P < 0.001.

The higher expression of STING in resistant model (BALB/c murine model) than in susceptible model (C57BL/6 murine model) at same time point after infection suggested that STING may play a potential protective role in PA keratitis. To explore the role of STING in PA keratitis, BALB/c mice were subconjunctivally injected with siSTING versus siNC, and then infected with PA. Clinical scores showed that silencing STING enhanced the disease severity at 3 and 5 days p.i. (both *P* < 0.05, Figure 2A). Respective slit photographs showed that siSTING-treated cornea exhibited corneal perforates with the grade of 4 at 5 days p.i. (siSTING, Figure 2B), while siNC-treated cornea displayed dense opacity covering the entire anterior segment with the grade of 3 at 5 days p.i. (siNC, Figure 2B). HE staining at 3 days p.i. showed that siSTING-treated corneas were much thicker and with more infiltrated inflammatory cells in the stroma and anterior chamber compared with siNC-treated corneas (Figure 2C). These data suggested that silencing STING accelerated the disease process of PA keratitis.

To investigate the reason why silencing STING deteriorated the disease process, inflammatory cytokines and bacterial load were measured by real-time PCR and bacterial plate count, respectively. PCR data showed that silencing STING promoted the expression of IL-1β (*P* < 0.01, Figure 2D), MIP-2 (*P* < 0.01, Figure 2F), TNF-α (*P* < 0.01, Figure 2G), and had no influence on IL-6 expression (Figure 2E). Moreover, bacterial plate count data showed that silencing STING elevated bacterial load (*P* < 0.001, Figure 2H). mRNA levels of STING, IFN-β, and ISG15 and protein levels of STING were detected by real-time PCR (Figure 2I) and Western blot (Figure 2J) to confirm the silencing efficacy. These data suggested that silencing STING promote the disease process of PA keratitis *via* enhancing inflammation cytokine expression and bacterial load.

### Activating STING Alleviated the Disease Process of PA Keratitis

**Figure 3:**
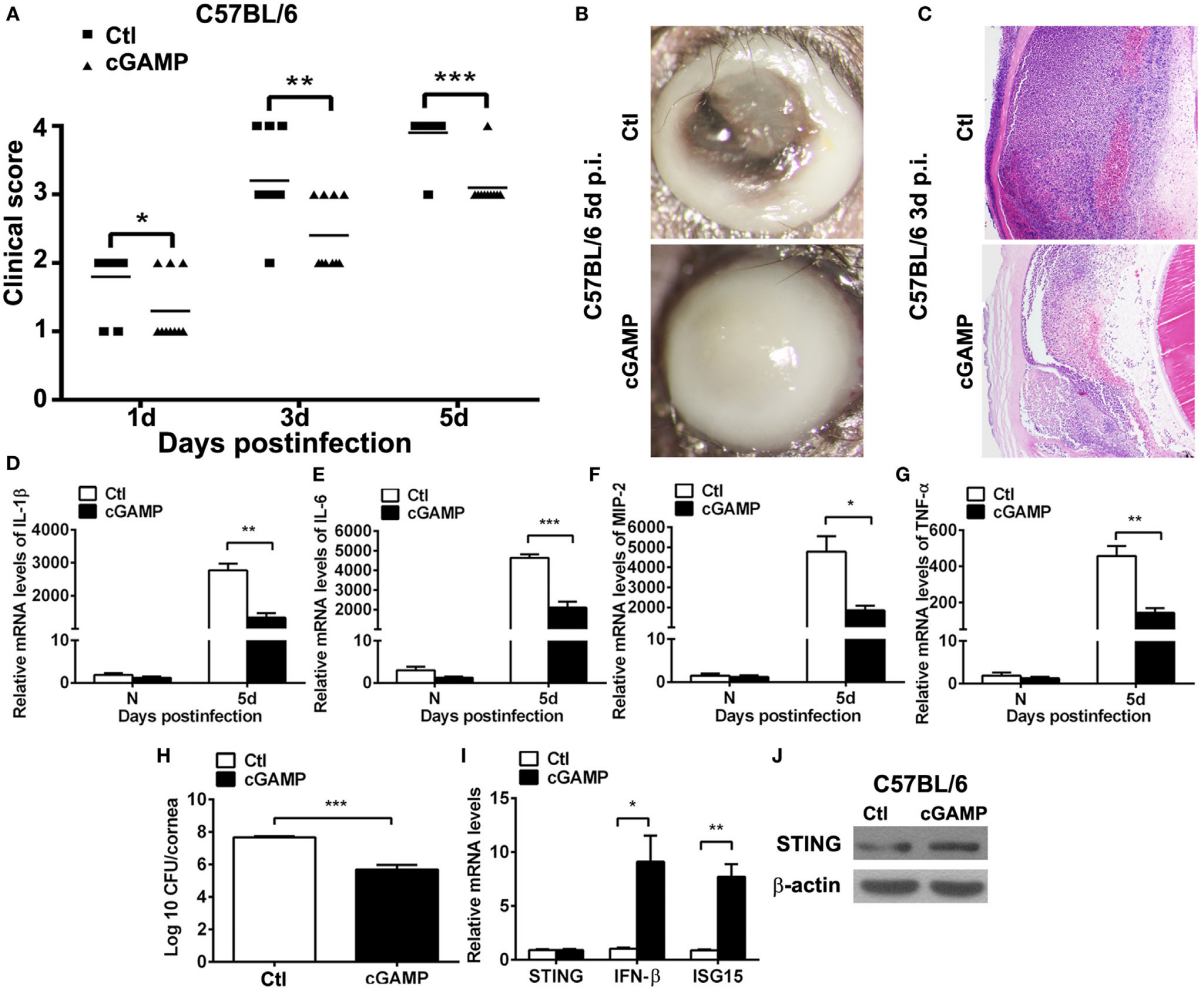
Activating stimulator of interferon genes (STING) alleviated the disease process of *Pseudomonas aeruginosa* (PA) keratitis. C57BL/6 mice were subconjunctivally injected with 2′,3′-cGAMP (cGAMP) versus control and then infected with PA routinely. Clinical scores **(A)** indicated the severity of the disease in cGAMP- versus control-treated groups. **(B)** Representative slit photographs of mouse corneas at 5 days postinfection (p.i.) displayed less opacity in cGAMP- versus control-treated mouse cornea (magnification ×10). **(C)** Hematoxylin–eosin staining was used to detect histopathology of infected cornea at 3 days p.i. in cGAMP- versus control-treated groups (magnification ×100). **(D–G)** mRNA levels of interleukin 1 beta (IL-1β) **(D)**, interleukin 6 (IL-6) **(E)**, macrophage inflammatory protein 2 (MIP-2) **(F)**, and tumor necrosis factor α (TNF-α) **(G)** were measured by using real-time PCR in normal and infected corneas at 5 days p.i. between cGAMP- and control-treated groups. **(H)** Bacterial load of infected cornea at 5 days p.i. was measured by plate count in cGAMP- versus control-treated groups. **(I)** mRNA levels of STING, IFN-β, and interferon-stimulated gene 15 (ISG15) and protein levels of STING **(J)** were measured by using real-time PCR and Western blot after treatment with cGAMP versus control. All data represent one of three independent experiments each using five pooled corneas per time. *P < 0.05; **P < 0.01; and ***P < 0.001.

To ascertain the role of STING in PA keratitis, C57BL/6 mice were subconjunctivally injected with the natural agonist of STING, cGAMP, and then infected with PA. Clinical scores showed that activating STING decreased the disease severity at 1, 3, and 5 days p.i. (*P* < 0.05, *P* < 0.01, and *P* < 0.001 at 1, 3, and 5 days, respectively; Figure 3A). Respective slit photos at 5 days p.i. showed that cGAMP-treated cornea exhibited dense opacity covering the entire anterior segment (grade = +3, cGAMP in Figure 3B), while control-treated mice displayed cornea perforates (grade = +4, Ctl in Figure 3B). HE staining at 3 days p.i. showed that cGAMP-treated corneas were much thinner and with less infiltrates compared with control-treated corneas (Figure 3C). These data suggested that activating STING alleviated the disease process of PA keratitis.

Furthermore, inflammatory cytokines and bacterial load were measured by real-time PCR and bacterial plate count, respectively. PCR data showed that activating STING suppressed the expression of IL-1β (*P* < 0.01, Figure 3D), IL-6 (*P* < 0.001, Figure 3E), MIP-2 (*P* < 0.05, Figure 3F), and TNF-α (*P* < 0.01, Figure 3G). Bacterial plate count data showed that activating STING enhanced bacterial elimination (*P* < 0.001, Figure 3H). mRNA levels of STING, IFN-β, and ISG15 and protein levels of STING were detected by real-time PCR (Figure 3I) and Western blot (Figure 3J) to confirm the activating efficacy. These data suggested that STING alleviated the disease process of PA keratitis *via* suppressing inflammation cytokine expression and bacterial load.

### Expression of STING *In Vitro* Macrophages

**Figure 4:**
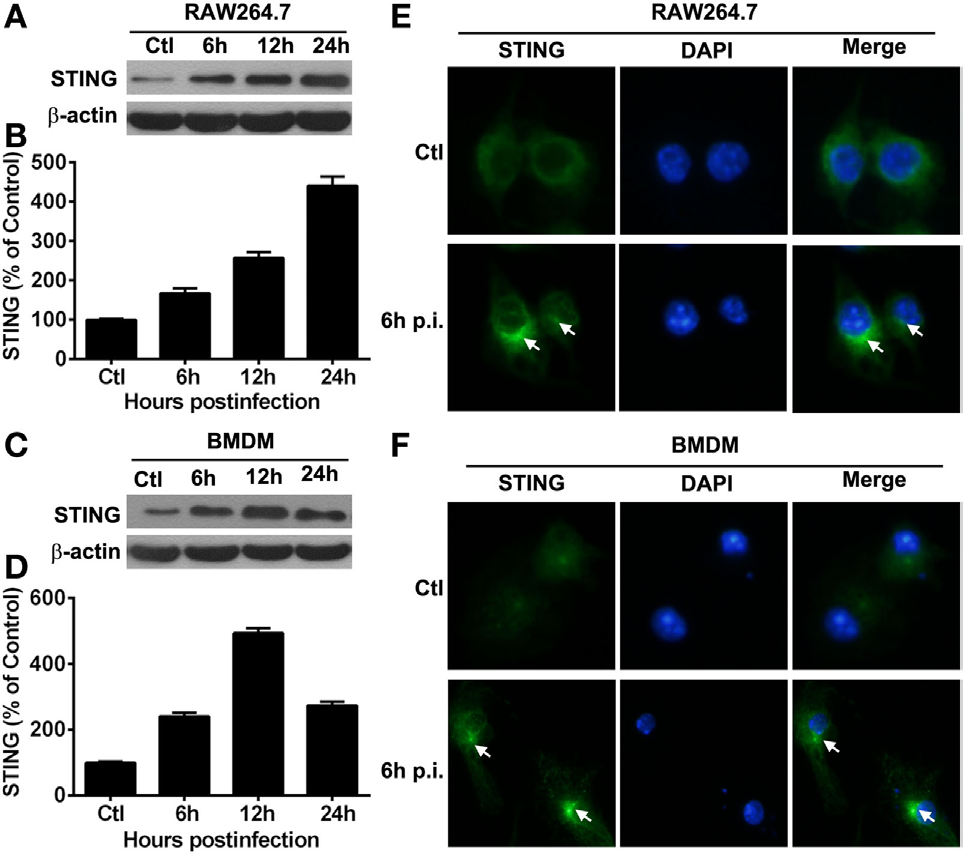
The expression of stimulator of interferon genes (STING) *in vitro* macrophages. **(A–D)** STING expression was determined by Western blot in murine macrophage-like RAW264.7 cells **(A)** and bone marrow-derived macrophages **(C)** at indicated time points after *Pseudomonas aeruginosa* (PA) infection. STING protein levels were quantitated by relative integrated density values after normalization to β-actin in murine macrophage-like RAW264.7 cells **(B)** and bone marrow-derived macrophages **(D)** Data were representative of three individual experiments. **(E,F)** STING expression and subcellular distribution (green staining) were measured by immunofluorescent in murine macrophage-like RAW264.7 cells **(E)** and bone marrow-derived macrophages **(F)** before and at 6 h after PA infection (white arrows indicate STING form perinuclear puncta, magnification = ×400).

Our *in vivo* data showed that STING expression was much higher in macrophages than in neutrophils, as indicated by flow cytometry, thus macrophages, rather than neutrophils, were used as the *in vitro* infectious model. To explore the activation of STING *in vitro*, the expression and subcellular location of STING in macrophages before and after PA infection were measured by Western blot and immunostaining, respectively. Western blot data showed that the protein levels of STING were upregulated at 6, 12, and 24 h p.i. in RAW264.7 cells (Figure 4A) and BMDM (Figure 4C), as indicated by the relative integrated density values (RAW264.7 cells shown in Figure 4B; BMDM shown in Figure 4D). Immunostaining data showed that STING was diffusely distributed in the cytosol in RAW264.7 cells (Ctl, Figure 4E) and BMDM (Ctl, Figure 4F) before infection, but aggregated at perinuclear area after infection in both cells (6 h p.i., Figures 4E,F). These data indicated that STING was activated in PA-infected macrophages.

### STING Suppressed the Expression of Inflammatory Cytokines *In Vitro*

**Figure 5:**
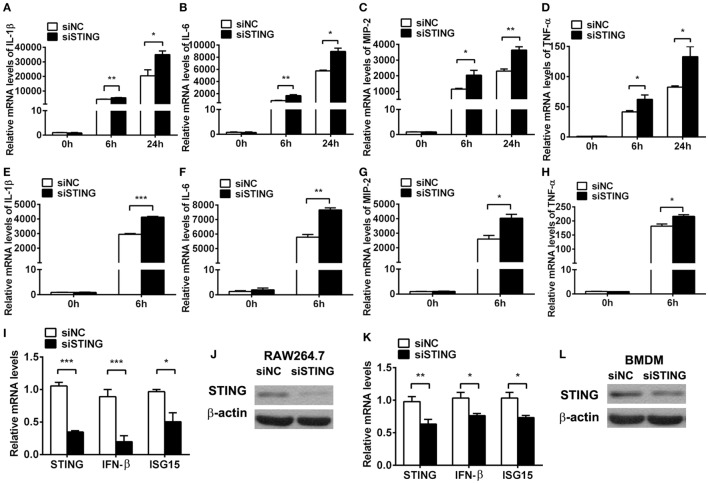
Silencing stimulator of interferon genes (STING) enhanced inflammatory cytokine expression. **(A–D)** mRNA levels of interleukin 1 beta (IL-1β) **(A)**, interleukin 6 (IL-6) **(B)**, macrophage inflammatory protein 2 (MIP-2) **(C)**, and tumor necrosis factor α (TNF-α) **(D)** were measured by using real-time PCR at 6 and 24 h postinfection (p.i.) in murine macrophage-like RAW264.7 cells with siRNA for mouse STING (siSTING) versus siNC treatment. **(E–H)** mRNA levels of IL-1β **(E)**, IL-6 **(F)**, MIP-2 **(G)**, and TNF-α **(H)** were measured by using real-time PCR at 6 h (p.i.) in bone marrow-derived macrophages with siSTING versus siNC treatment. **(I,K)** mRNA levels of STING, IFN-β, and interferon-stimulated gene 15 (ISG15) were measured by using real-time PCR in RAW264.7 cells **(I)** and bone marrow-derived macrophage (BMDM) **(K)** with siSTING versus siNC treatment. **(J,L)** Protein levels of STING were measured by using Western blot in RAW264.7 cells **(J)** and BMDM **(L)** with siSTING versus siNC treatment. Data are shown as mean ± SEM of three independent experiments. *P < 0.05; **P < 0.01; and ***P < 0.001.

To ascertain the role of STING in regulating inflammation, *in vitro* macrophages were used to detect inflammatory cytokine expression after silencing and activating STING. PCR data showed that silencing STING enhanced the expression of IL-1β (*P* < 0.01 and *P* < 0.05, at 6 and 24 h, respectively, Figure 5A), IL-6 (*P* < 0.01 and *P* < 0.05, at 6 and 24 h, respectively, Figure 5B), MIP-2 (*P* < 0.05 and *P* < 0.01, at 6 and 24 h, respectively, Figure 5C), and TNF-α (both *P* < 0.05, at 6 and 24 h, Figure 5D) at 6 and 24 h p.i. in RAW264.7 cells; meanwhile, silencing STING increased the expression of IL-1β (*P* < 0.001, Figure 5E), IL-6 (*P* < 0.01, Figure 5F), MIP-2 (*P* < 0.05, Figure 5G), and TNF-α (*P* < 0.05, Figure 5H) in BMDM. The silencing efficacy was confirmed by detecting the mRNA levels of STING, IFN-β, and ISG15 (Figures 5I,K) and the protein levels of STING (Figures 5J,L) in RAW264.7 cells (Figures 5I,J) and BMDM (Figures 5K,L).

**Figure 6:**
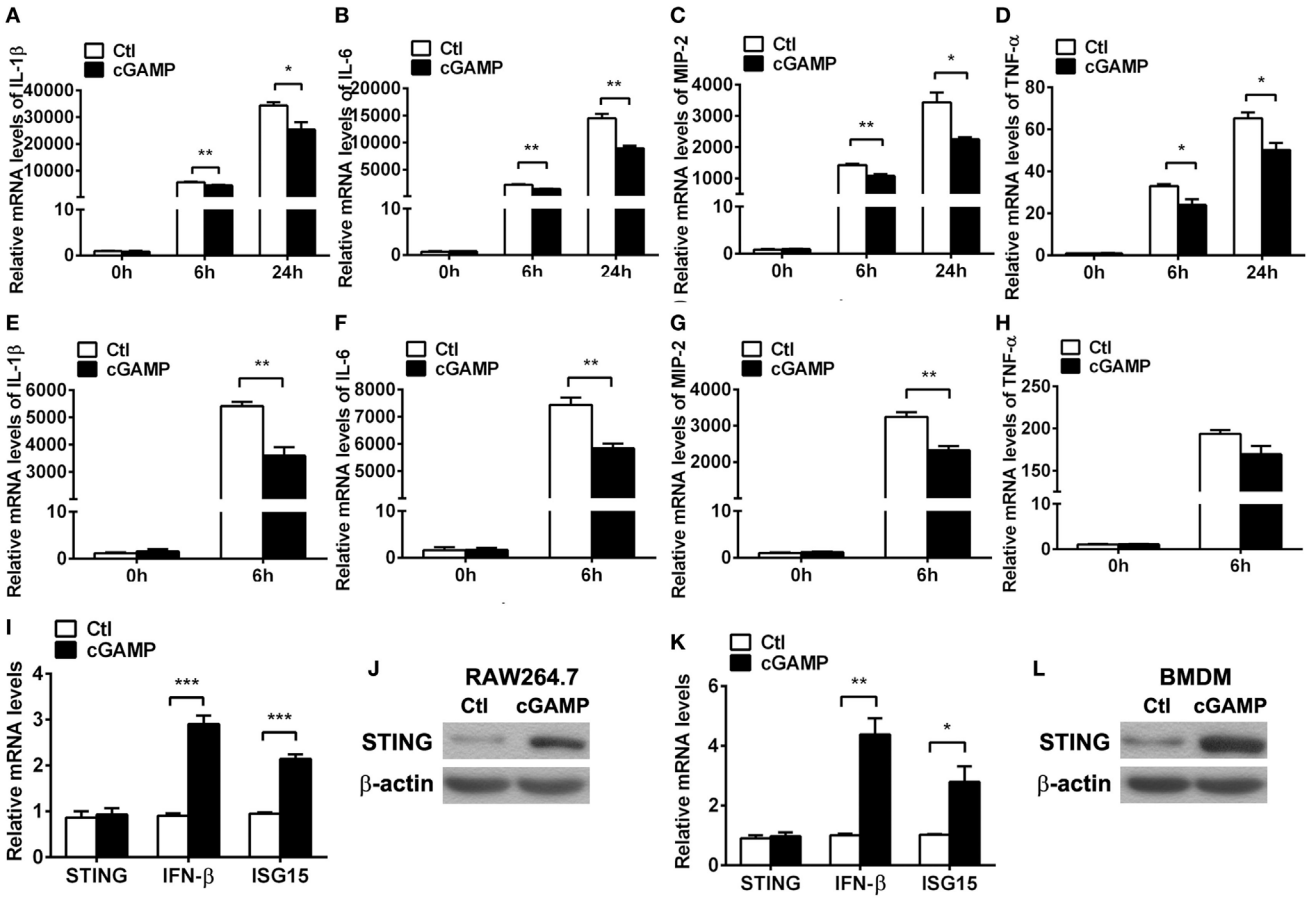
Activating stimulator of interferon genes (STING) inhibited inflammatory cytokine expression. **(A–D)** mRNA levels of interleukin 1 beta (IL-1β) **(A)**, interleukin 6 (IL-6) **(B)**, macrophage inflammatory protein 2 (MIP-2), **(C)** and tumor necrosis factor α (TNF-α) **(D)** were measured by using real-time PCR at 6 and 24 h postinfection (p.i.) in murine macrophage-like RAW264.7 cells with 2′,3′-cGAMP (cGAMP) versus control treatment. **(E–H)** mRNA levels of IL-1β **(E)**, IL-6 **(F)**, MIP-2 **(G)**, and TNF-α **(H)** were measured by using real-time PCR at 6 h p.i. in bone marrow-derived macrophages with cGAMP versus control treatment. **(I,K)** mRNA levels of STING, IFN-β, and interferon-stimulated gene 15 (ISG15) were measured by using real-time PCR in RAW264.7 cells **(I)** and bone marrow-derived macrophage (BMDM) **(K)** with cGAMP versus control treatment. **(J,L)** Protein levels of STING were measured by using Western blot in RAW264.7 cells **(J)** and BMDM **(L)** with cGAMP versus control treatment. Data are shown as mean ± SEM of three independent experiments. *P < 0.05; **P < 0.01; and ***P < 0.001.

Furthermore, cGAMP was used to activate STING to confirm the regulation role of STING in the inflammatory cytokine expression *in vitro*. PCR data showed that activating STING suppressed the mRNA levels of IL-1β (*P* < 0.01 and *P* < 0.05, at 6 and 24 h, respectively, Figure 6A), IL-6 (both *P* < 0.01, at 6 and 24 h, Figure 6B), MIP-2 (*P* < 0.01 and *P* < 0.05, at 6 and 24 h, respectively, Figure 6C), and TNF-α (both *P* < 0.05, at 6 and 24 h, Figure 6D) at 6 and 24 h p.i. in RAW264.7 cells; meanwhile, activating STING decreased the expression of IL-1β (*P* < 0.01, Figure 6E), IL-6 (*P* < 0.01, Figure 6F), and MIP-2 (*P* < 0.01, Figure 6G), but had no influence on TNF-α expression (Figure 6H) in BMDM. The activating efficacy was confirmed by detecting the mRNA levels of STING, IFN-β, and ISG15 (Figures 6I,K) and the protein levels of STING (Figures 6J,L) in RAW264.7 cells (Figures 6I,J) and BMDM (Figures 6K,L). These data together demonstrated that STING suppressed the PA-induced expression of inflammatory cytokines *in vitro* infection model.

### STING Enhanced Bacterial Elimination *In Vitro*

**Figure 7:**
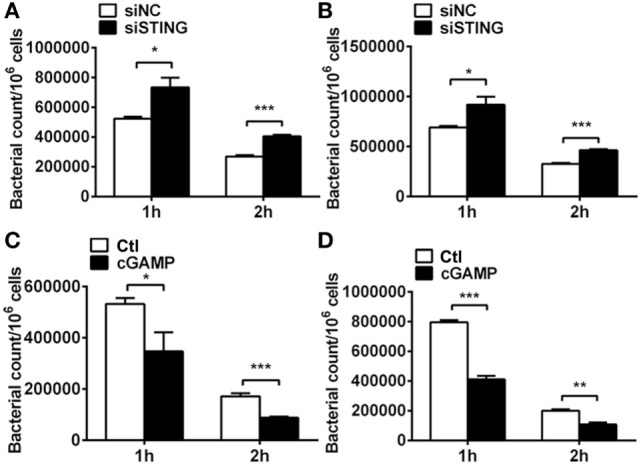
Stimulator of interferon genes (STING) regulated bacterial elimination *in vitro*. **(A,B)** Bacterial plate count was used to measure intracellular bacterial killing in murine macrophage-like RAW264.7 cells **(A)** and bone marrow-derived macrophages **(B)** with siRNA for mouse STING (siSTING) versus siNC treatment. **(C,D)** Bacterial plate count was used to measure intracellular bacterial killing in murine macrophage-like RAW264.7 cells **(C)** and bone marrow-derived macrophages **(D)** with 2′,3′-cGAMP (cGAMP) versus control treatment. Data are shown as mean ± SEM of three independent experiments. *P < 0.05; **P < 0.01; and ***P < 0.001.

Pathogenesis of PA keratitis depends largely on the tissue damage caused by excessive inflammatory response, as well as bacteria invasion. Therefore, we detected bacterial elimination *in vitro* by using plate count assay. Data showed that silencing STING inhibited bacterial killing at 1 and 2 h p.i. in RAW264.7 cells (*P* < 0.05 and *P* < 0.001, respectively, Figure 7A) and BMDM (*P* < 0.05 and *P* < 0.001, respectively, Figure 7B), whereas activating STING enhanced bacterial clearance at 1 and 2 h p.i. in RAW264.7 cells (*P* < 0.05 and *P* < 0.001, respectively, Figure 7C) and BMDM (*P* < 0.001 and *P* < 0.01, respectively, Figure 7D). These data together suggested that STING promoted bacterial killing *in vitro*.

### STING Suppressed Inflammatory Cytokine Expression *via* Restraining NF-κB Activation

**Figure 8:**
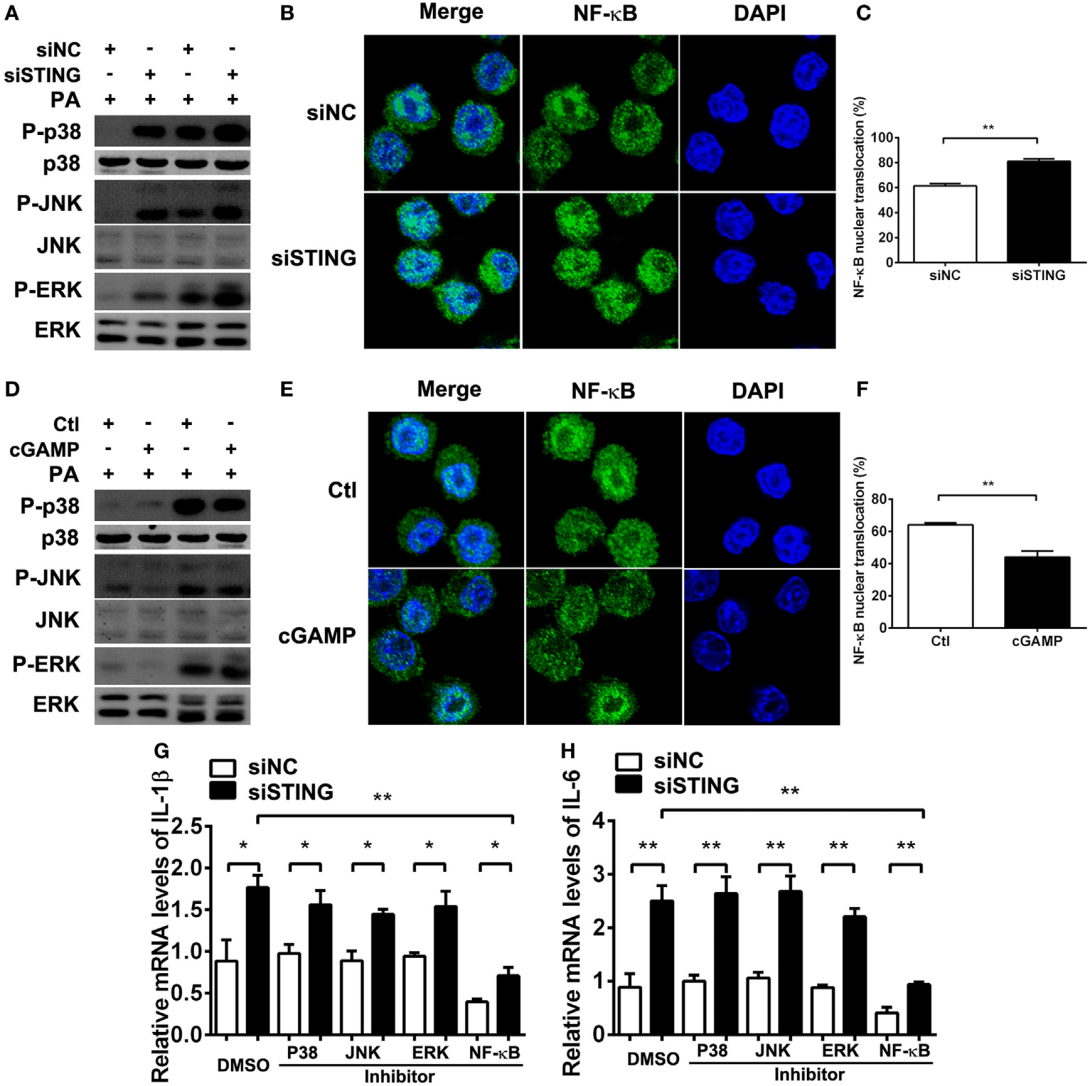
Stimulator of interferon genes (STING) suppressed inflammatory cytokine expression *via* restraining nuclear factor-κB (NF-κB) activity. **(A,D)** The protein levels of phosphorylated p38 (P-p38), p38, phosphorylated c-Jun N-terminal kinase (P-JNK), c-Jun N-terminal kinase (JNK), phosphorylated extracellular regulated protein kinases (P-ERK), and ERK were measured by Western blot at 30 min and 1 h postinfection (p.i.) in RAW264.7 cells after being treated with siRNA for mouse STING (siSTING) versus siNC **(A)** and 2′,3′-cGAMP (cGAMP) versus control **(D)**. **(B,E)** The nuclear translocation of NF-κB (the colocalization of NF-κB and nuclear staining with 4,6-diamino-2-phenyl indole) was measured by immunofluorescence at 6 h p.i. in RAW264.7 cells after being treated with siSTING versus siNC **(B)** and cGAMP versus control **(E)**, and **(C,F)** the percentage of cells with NF-κB nuclear translocation was quantified by counting more than 200 cells in three random fields. **(G,H)** mRNA levels of interleukin 1 beta (IL-1β) **(G)** and interleukin 6 (IL-6) **(H)** were measured by real-time PCR at 6 h p.i. in RAW264.7 cells after treatment with siSTING versus siNC, followed by treatment with P38 inhibitor, JNK inhibitor, ERK inhibitor, NF-κB inhibitor versus DMSO vehicle control. Data are shown as mean ± SEM of three independent experiments. *P < 0.05; **P < 0.01.

Previous studies demonstrated that STING-induced type I IFN suppressed immune responses in chronic infection. To explore whether the anti-inflammatory role of STING in PA infection depends on type I IFN, we used cGAMP to activate STING-type I IFN signaling, followed by using anti-IFN-β antibody to block type I IFN signaling. PCR data showed that activating STING suppressed the expression of IL-1β (Figure S1A in Supplementary Material) and IL-6 (Figure S1B in Supplementary Material) at 6 and 24 h p.i. in RAW264.7 cells, which was consistent with our former data. However, blocking type I IFN signaling could not reverse the anti-inflammatory role of STING (Figures S1A,B in Supplementary Material). These data suggested that the inhibitory effect of STING on inflammation was independent of type I IFN. To further explore the anti-inflammatory mechanism underlying, the activity of TLR downstream signaling molecules including MAPK and NF-κB were detected by Western blot and immunofluorescence, respectively. The data showed that silencing STING upregulated the phosphorylation of p38, JNK, and ERK (Figure 8A) and promoted nuclear translocation of NF-κB (Figures 8B,C), whereas activating STING downregulated the phosphorylation of p38, JNK, and ERK (Figure 8D) and suppressed nuclear translocation of NF-κB (Figures 8E,F). Although these data indicated that STING regulated the activity of both MAPK and NF-κB, our following PCR data showed that only NF-κB inhibitor, but not p38, JNK, and ERK inhibitors reversed the increasing expression of IL-1β (*P* < 0.01, Figure 8G) and IL-6 (*P* < 0.01, Figure 8H) after silencing STING. Therefore, these data indicated that STING suppressed inflammatory cytokine expression *via* restraining NF-κB activity.

### STING-Induced iNOS Expression

**Figure 9:**
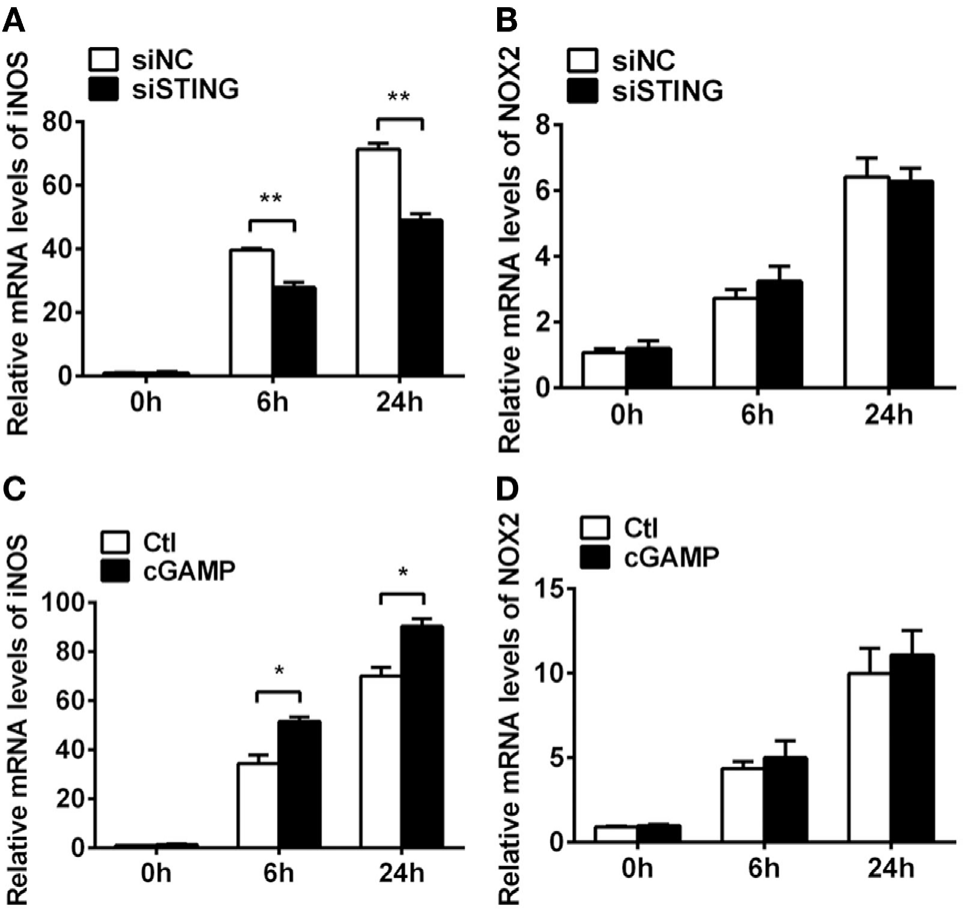
Stimulator of interferon genes (STING) induced inducible NO synthase (iNOS) expression. **(A,B)** mRNA levels of iNOS **(A)** and nicotinamide adenine dinucleotide phosphate oxidase 2 (NOX2) **(B)** were measured by using real-time PCR at 6 h and 24 h postinfection (p.i.) in RAW264.7 cells after treatment with siRNA for mouse STING (siSTING) versus siNC. **(C,D)** mRNA levels of iNOS **(C)** and NOX2 **(D)** were measured by using real-time PCR at 6 and 24 h p.i. in RAW264.7 cells after treatment with 2′,3′-cGAMP (cGAMP) versus control. Data are shown as mean ± SEM of three independent experiments. *P < 0.05; **P < 0.01.

Previous studies demonstrated that STING-induced type I IFN promoted host resistance against various virus and bacterial infections. To determine the role of type I IFN in bacterial killing after PA infection, bacteria plate count assay was measured and the data showed that blocking IFN signaling could not reverse the STING-induced bacterial killing ability (Figure S1C in Supplementary Material). Moreover, the bactericidal mechanism including iNOS and NOX2 was measured by real-time PCR, and the data showed that silencing STING inhibited iNOS expression at 6 h (*P* < 0.01, Figure 9A) and 24 h p.i. (*P* < 0.01, Figure 9A) in RAW264.7 cells, whereas activating STING promoted iNOS expression at 6 h (*P* < 0.05, Figure 9C) and 24 h p.i. (*P* < 0.05, Figure 9C) in RAW264.7 cells. However, STING had no influence on NOX2 expression (Figures 9B,D). These data indicated that STING promoted bacterial clearance *via* enhancing iNOS expression.

## Discussion

As a central molecule of cytosolic DNA sense signaling, STING plays a critical role in various physiological and pathological processes, especially in bacteria and virus infections (10–19). However, the function of STING in modulating PA-infected corneal disease remains unclear. Our study demonstrated that STING reduced the severity of PA keratitis by decreasing corneal inflammation and enhancing bacterial clearance, which shed some light on the regulatory mechanism of ocular infection.

Previously, tremendous pathogens such as DNA virus (10–12), retrovirus (13), intracellular bacteria (14–16, 18, 19), and extracellular bacteria (17) can stimulate STING signaling. So far, little is known regarding the activation of STING in response to PA infection. Our *in vivo* data showed that the protein levels of cGAS, P-STING, and STING, as well as IFN-β gene expression was upregulated in PA-infected mouse corneas, suggesting the activation of STING in PA-infected cornea. To clarify the cell source of STING, we detected the STING expression in the corneal filtrating macrophages and neutrophils using flow cytometry. Data demonstrated that STING was mainly expressed in F4/80+ macrophages, rather than Gr-1+ neutrophils, which is consistent with previous study showing that STING is absent in Ly6G+ neutrophils (19). Thus, in the following *in vitro* study, we used murine macrophage-like RAW264.7 cells and BMDM to explore the expression and function of STING in response to PA infection. *In vitro* expression data also showed that STING was upregulated, activated, and formed perinuclear puncta in PA-stimulated macrophages, which supported our *in vivo* observation that STING was activated in PA keratitis.

Stimulator of interferon genes was reported to mediate host resistance to HSV-1 infection at the ocular surface (30, 31). Our *in vivo* and *in vitro* silencing and activating studies also demonstrated that STING promoted host resistance against PA keratitis, which might be relevant to the vaccine adjuvant character of STING agonist (32). Furthermore, our *in vivo* and *in vitro* data indicated that STING decreased the stromal infiltration of immune cells and the production of inflammatory cytokines including IL-1β, IL-6, MIP-2, and TNF-α, suggesting that STING plays an anti-inflammatory function in PA-induced keratitis. It is reported that STING-induced type I IFN plays an anti-inflammatory function *via* suppressing Th1 immune responses (33) or IL-1β secretion (34) in chronic infection. However, in acute bacterial infection, type I IFN might not be pivotal in exerting anti-inflammatory effect. Our data found that the anti-inflammatory role of STING in PA infection was not influenced by blocking type I IFN signaling, which excluded the inhibitory effect of STING-induced type I IFN on PA-induced inflammation. Previous studies elucidated that STING suppressed inflammation by interfering TLR signaling. Sharma et al. demonstrated that STING-deficient macrophages were hyperresponsive to TLR ligands, with lack of negative regulators of TLR signaling (26). Our data showed that STING did not affect the mRNA levels of TLR2/4/5/9 (data not shown), but regulated the activation of TLR downstream molecules including the phosphorylation of MAPK and the nuclear translocation of NF-κB; further data found that STING suppressed inflammatory cytokine secretion *via* inhibiting NF-κB activity.

The role of STING in regulating pathogen elimination was diversity according to the type of pathogens and different research models (10–19). STING enhances microbe clearance when infected with HSV (10, 11), CMV (12), HIV (13), and *M. tuberculosis* (14, 15), but facilitates bacteria escape during *Brucella* species (16) and *S. aureus* (17) infection. Besides, STING plays a controversial role in *L. monocytogenes* infection because of different research models (18, 19). However, till now the role of STING in PA clearance remains unknown. Our *in vivo* and *in vitro* data showed that STING enhanced bacterial elimination in PA infection. Substantial evidence demonstrated that STING-induced type I IFN is critical in host resistance against virus (10–13) and intracellular bacterial infection (15). However, our data showed that STING enhanced bacterial killing *via* promoting iNOS expression, an oxygen-dependent bactericidal mechanism, but not type I IFN.

Inflammatory cytokines are often beneficial for bacterial elimination (2). However, our data demonstrated that STING suppressed the inflammatory cytokine expression, but enhanced bacterial killing, which seemed to be contradictory with each other. The result could be explained by two possible mechanisms. First, STING promoted iNOS production, which is a critical bactericidal mechanism in PA infection (35). Second, amplified inflammation causes tissue damage, which is adverse to bacterial clearance (2). Therefore, STING might participate in restricting uncontrolled inflammation and ultimately be beneficial for anti-infection immunity.

In conclusion, our data indicated that STING promoted host resistance against PA keratitis by restricting corneal inflammatory response and bacterial killing. These data uncovered the protective role of STING in infected immune response and host–pathogen interaction, which may provide a potential therapy for PA keratitis.

## Ethics Statement

This study was carried out in accordance with the guidelines of Animal Care and Use of Sun Yat-sen University. The protocol was approved by Sun Yat-sen University.

## Author Contributions

KC, QF, XW, and MW wrote the manuscript. MW designed experiments. KC, QF, SL, YL, WQ, YoW, and XW performed experiments and analyzed data. LW, YoW, and YiW provided scientific expertise. MW and WW supervised the project.

## Conflict of Interest Statement

The authors declare that the research was conducted in the absence of any commercial or financial relationships that could be construed as a potential conflict of interest.

## Supplementary Material

Figure S1 Stimulator of interferon genes (STING) suppressed inflammatory cytokine expression and promoted bacterial killing independent of type I IFN. **(A,B)** mRNA levels of interleukin 1 beta (IL-1β) **(A)** and interleukin 6 (IL-6) **(B)** were measured by real-time PCR at 6 and 24 h postinfection (p.i.) in RAW264.7 cells after treatment with 2′,3′-cGAMP (cGAMP) versus control, followed by treatment with IgG versus anti-IFN-β antibody. **(C)** Bacterial elimination was measured by bacterial plate count assay at 1 and 2 h p.i. in RAW264.7 cells after treatment with cGAMP versus control, followed by treatment with IgG versus anti-IFN-β antibody. **(D)** mRNA levels of interferon-stimulated gene 15 (ISG15) were measured by real-time PCR in RAW264.7 cells treated with anti-IFN-β antibody versus IgG, to ensure the blocking efficacy of type I IFN signaling. Data are shown as mean ± SEM of three independent experiments. **P* < 0.05; ***P* < 0.01; and ****P* < 0.01.
