# Supplementary figures and images for: Stimulator of Interferon Genes Promotes Host Resistance Against Pseudomonas aeruginosa Keratitis
Source: Front Immunol. 2018 Jun 5;9:1225. doi: 10.3389/fimmu.2018.01225 (PMC5996077; doi:10.3389/fimmu.2018.01225)

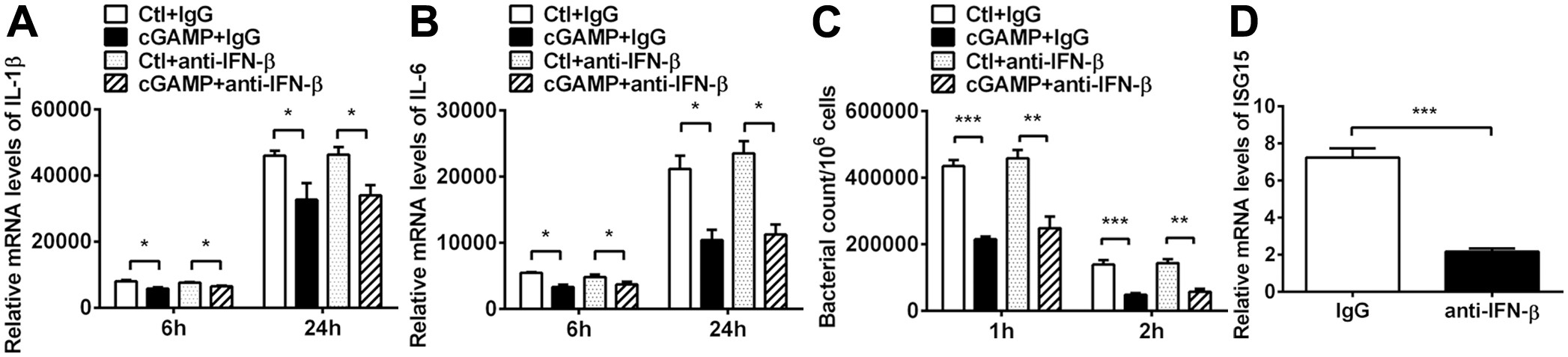

Supplement: Figure S1 — Stimulator of interferon genes (STING) suppressed inflammatory cytokine expression and promoted bacterial killing independent of type I IFN. (A,B) mRNA levels of interleukin 1 beta (IL-1β) (A) and interleukin 6 (IL-6) (B) were measured by real-time PCR at 6 and 24 h postinfection (p.i.) in RAW264.7 cells after treatment with 2′,3′-cGAMP (cGAMP) versus control, followed by treatment with IgG versus anti-IFN-β antibody. (C) Bacterial elimination was measured by bacterial plate count assay at 1 and 2 h p.i. in RAW264.7 cells after treatment with cGAMP versus control, followed by treatment with IgG versus anti-IFN-β antibody. (D) mRNA levels of interferon-stimulated gene 15 (ISG15) were measured by real-time PCR in RAW264.7 cells treated with anti-IFN-β antibody versus IgG, to ensure the blocking efficacy of type I IFN signaling. Data are shown as mean ± SEM of three independent experiments. *P < 0.05; **P < 0.01; and ***P < 0.01. [file image_1.TIF]
